# Potent PDE4 inhibitor activates AMPK and Sirt1 to induce mitochondrial biogenesis

**DOI:** 10.1371/journal.pone.0253269

**Published:** 2021-06-17

**Authors:** Sung-Jun Park, Faiyaz Ahmad, Robert J. Bahde, Andrew Philp, Jeonghan Kim, Tianjiao Huang, Myung K. Kim, William C. Trenkle, Jay H. Chung

**Affiliations:** 1 Laboratory of Obesity and Aging Research, Cardiovascular Branch, National Heart Lung and Blood Institute, National Institutes of Health, Bethesda, Maryland, United States of America; 2 Department of Pediatric Medicine, Division of Endocrinology, Sidra Medical and Research Center, Doha, Qatar; 3 Laboratory of Cell and Molecular Biology, National Institute of Diabetes and Digestive and Kidney Diseases, National Institutes of Health, Bethesda, Maryland, United States of America; 4 Mitochondrial Metabolism and Ageing Laboratory, Diabetes and Metabolism Division, Garvan Institute of Medical Research, Darlinghurst, Australia; Georgia State University, UNITED STATES

## Abstract

AMP-activated protein kinase (AMPK) is an evolutionarily conserved energy sensor. Activation of AMPK leads to a number of metabolic benefits, including improved mitochondrial function in skeletal muscle and lowering of serum glucose levels in type-2 diabetes models. However, direct activation of AMPK leads to cardiac enlargement, and an alternative strategy that activates AMPK without affecting the heart is needed. Inhibition of phosphodiesterase 4 (PDE4), which is poorly expressed in the human heart, activates AMPK in other tissues. In a screen to identify novel PDE4 inhibitors, we discovered compound CBU91, which is 5–10 fold more potent than rolipram, the best characterized PDE4 inhibitor. CBU91, like rolipram, is able to activate AMPK and Sirt1 and increase mitochondrial function in myotubes. These findings suggest that activation of AMPK in myotubes is a general property of PDE4 inhibition and that PDE4 inhibition may activate AMPK in metabolically relevant tissues without affecting the heart.

## Introduction

Intracellular cyclic adenosine monophosphate (cAMP) levels are tightly controlled by adenylate cyclases (ACs), which catalyze the cyclization of adenosine triphosphate (ATP) to cAMP, and phosphodiesterases (PDEs), which degrade cAMP by hydrolyzing the phosphodiester bond of cAMP to generate AMP [1]. The PDE family is composed of eleven members: PDEs 4, 7 and 8 selectively hydrolyze cAMP; PDEs 1, 2, 3, 10, and 11 hydrolyze both cAMP and cGMP; PDEs 5, 6 and 9 hydrolyze cGMP. PDE4, the largest member of the mammalian PDE family, is encoded by four genes, PDE4 A to D, which together produce more than 25 splice variants. PDE4 isotypes are widely expressed throughout the whole body, but are highly expressed in skeletal muscle as well as in neural, immune and inflammatory cells, but not expressed or poorly expressed in the heart [1–4].

PDE4 may be a target for treating a number of medical conditions [5–7]. Rolipram, the most widely studied general PDE4 inhibitor, has been shown to have antidepressant properties and to improve cognition and memory in animal models [8, 9]. In addition, PDE4 inhibitors exhibit anti-inflammatory properties [10–13]. Rolipram can also mimic the metabolic effects of the polyphenol resveratrol by activating AMP-dependent kinase (AMPK) and increasing mitochondrial function [14], thereby protecting mice on a high fat diet against obesity and improving glucose tolerance [15, 16]. However, it is not known whether the ability to activate AMPK and Sirt1 in myotubes is unique to rolipram or is a general property of PDE4 inhibitors.

As part of our ongoing studies into the cellular roles and functions of PDE4, AMPK and Sirt1, we screened a set of novel compounds for PDE4 inhibitory activity. We have identified a lead compound, CBU91, which is 5–10 fold more potent than rolipram in inhibition of PDE4 and has the ability to activate AMPK and increase mitochondrial function in myotubes.

## Materials and methods

### General information

General experimental information has been previously reported [17].

^1^H NMR spectra are reported in ppm relative to residual protiated solvent (CDCl_3_: 7.26 ppm, (CD_3_)_2_SO: 2.50 ppm) and ^13^C NMR spectra are reported in ppm relative to residual protiated solvent (CDCl_3_: 77.23 ppm, (CD_3_)_2_SO: 39.50 ppm). ^1^H NMR spectra were obtained at 300 or 400 MHz and ^13^C NMR spectra were obtained at 100 MHz. Data are presented as follows: chemical shift, multiplicity (s = singlet, d = doublet, t = triplet, q = quartet, m = multiplet, br = broad), integration, and coupling constants (in Hertz).

### Synthetic procedures

#### Amide formation

3,4-dimethoxy-*N*-(*o*-tolyl)benzamide: A solution of commercially available potassium 2-methylbutan-2-olate (12.78 ml, 21.73 mmol, 1.09 equiv) in toluene (1.7 M) via syringe was added to a 90 °C solution of *o*-toluidine (2.36 g, 22.0 mmol, 1.10 equiv) in toluene (20 ml). The reaction mixture was refluxed for 30 min, then a solution of 3,4-dimethoxybenzoyl chloride (4.00 g, 20.0 mmol, 1.00 equiv) in toluene (20 ml) was added dropwise via syringe to the refluxing solution. The mixture was refluxed for an additional hour followed by a second addition of potassium 2-methylbutan-2-olate (12.78 ml, 21.73 mmol, 1.09 equiv) in toluene (1.7 M). The mixture was refluxed for 4 h. The reaction was cooled to room temperature overnight. The resulting mixture was heated to 80 °C followed by sequential addition of water (200 mL) and concentrated HCL (14 mL). The bottom aqueous layer was removed. The organic layer contained a suspended solid. The organic layer was filtered to afford 3,4-dimethoxy-N-o-tolylbenzamide (CBU-91, 2.46 g, 14.74 mmol, 73.9% yield) as a gray solid.

^1^H NMR (400 MHz, CDCl_3_) δ 7.95 (d, 1H, *J* = 8.3Hz), 7.63 (s, 1H), 7.54 (d, 1H, *J* = 2.0Hz), 7.41 (dd, 1H, *J* = 8.3, 2.1Hz), 7.23–7.29 (m, 1H), 7.10–7.15 (m, 1H), 6.93 (d, 1H *J* = 8.4Hz), 3.97 (s, 3H), 3.96 (s, 3H), 2.35 (s, 3H); ^13^C NMR (100 MHz, CDCl_3_) δ 165.6, 152.5, 149.3, 137.6, 134.8, 131.2, 127.7, 127.6, 126.9, 126.8, 121.9, 111.9, 111.9, 56.6, 56.5, 18.9; HRMS (ESI) *m*/*z* 272.1380 (272.1281 calc for C_16_ H_18_ O_3_ N_1_, MH).

#### *N*-(3,5-dichloropyridin-4-yl)-3,4,5-trimethoxybenzamide (CBU-090)

3,5-Dichloropyridin-4-amine (0.995 g, 6.10 mmol) and 3,4,5-trimethoxybenzoyl chloride (1.28 g, 5.55 mmol) provided 1.38 g of *N*-(3,5-dichloropyridin-4-yl)-3,4,5-trimethoxybenzamide (70%) as a white solid.

^1^H NMR (400 MHz, CDCl_3_) δ 8.57 (s, 2H), 7.74 (br s, 1H), 7.19 (s, 2H), 3.94 (s, 6H), 3.93 (s, 3H); ^13^C NMR (100 MHz, CDCl_3_) δ 165.1, 153.7, 149.3, 142.3, 142.0, 131.7, 128.5, 106.5, 61.1, 57.1; HRMS (ESI) *m*/*z* 357.0398 (357.0403 calc for C_15_ H_15_ O_6_ N_2_ Cl_2_, MH).

#### *N*-(*o*-tolyl)quinoxaline-2-carboxamide (CBU-92)

*o*-Toluidine (0.092 g, 0.857 mmol) and quinoxaline-2-carbonyl chloride (0.15 g, 0.779 mmol) provided 0.080g of *N*-(*o*-tolyl)quinoxaline-2-carboxamide (39.0%) as an off-white solid.

^1^H NMR (400 MHz, DMSO) δ 10.44 (br s, 1H), 9.57 (s, 1H), 8.30–8.23 (m, 2H), 8.05–8.01 (m, 2H), 7.72 (d, 1H, *J =* 7.6Hz), 7.34–7.25 (m, 2H), 7.19 (t, 1H, *J* = 7.5Hz), 2.36 (s, 3H); ^13^C NMR (100 MHz, CDCl_3_) δ 162.5, 145.2, 144.7, 144.0, 140.6, 136.6, 133.1, 132.7, 132.4, 131.4, 130.6, 130.1, 127.2, 126.7, 125.3, 18.6; HRMS (ESI) *m*/*z* 264.1129 (264.1131 calc for C_16_ H_14_ O_1_ N_3_, MH).

#### Hydrazone formation

*N’*-[4-(diethylamino)-2-hydroxybenzylidene]-2-hydroxybenzohydrazide (CBU-93): Salicyloyl hydrazide and 4-(diethylamino)salicylaldehyde were combined in glacial acetic acid at room temperature. The product of condensation precipitated to provide N’-[4-(diethylamino)-2-hydroxybenzylidene]-2-hydroxybenzohydrazide in quantitative yield after filtration. This compound is commercially available.

^13^C NMR (100 MHz, CDCl_3_) δ 165.0, 160.7, 160.3, 151.7, 151.3, 134.7, 132.6, 129.1, 119.8, 118.3, 116.2, 107.3, 104.7, 98.4, 44.8, 13.5; HRMS (ESI) *m*/*z* 328.1658 (328.1656 calc for C_18_ H_22_ O_3_ N_3_, MH).

### Cell culture

C2C12 myoblast cells (ATCC) were maintained in DMEM and 10% fetal bovine serum. To generate C2C12 myotubes, confluent cultures of C2C12 cells were grown in DMEM with 2% horse serum for 3–5 days.

### Cyclic AMP measurement

The cyclic AMP complete enzyme immunoassay kit from Enzo Life Sciences was used as directed by the manufacturer.

### Ca^2+^ signal measurements

C2C12 myotubes were preincubated with 20 μM U73122 for 1 hr. Ca^2+^ release was measured using the fluorescent calcium indicator Fluo-4AM (Molecular Probes) according to the manufacturer`s suggestions.

### Mitochondrial DNA (mtDNA) quantification

Relative amounts of nuclear DNA and mtDNA were determined by quantitative Real-Time PCR. Differentiated C2C12 myotubes were incubated with Proteinase K overnight in a lysis buffer for DNA extraction by using the DNeasy kit (QIAGEN). Quantitative PCR was performed by using the following primers (mtDNA, forward 5`-CCGCAAGGGAAAGATGAAAGA-3`, reverse 5`-TCGTTTGGTTTCGGGGTTTC-3`; and nuclear DNA, forward 5`-GCCAGCCTCTCCTGATGT-3`, reverse 5`- GGGAACACAAAAGACCTCTTCTGG-3` and FastStart Universal SYBR Green Master mix in a LightCycler 96 (Roche) with a program of 20 minutes at 95°C, followed by 50 to 60 cycles of 15 seconds at 95°C, 20 seconds at 58°C and 20 seconds at 72°C. Mitochondrial DNA content was normalized with nuclear DNA content.

### Immunoblotting

Cells were lysed in RIPA buffer and subjected to immunoblotting. The following antibodies were used: AMPK, p-AMPK (T172), p-ACC (S79), ACC, p-CREB (S133), p-PKAc (T197), GAPDH, LC3, p62 (Cell Signaling Technology) and Actin (Santa Cruz). PGC-1α acetylation was visualized by immunoprecipitation from the cell extract (500 μg) using Flag antibody (Sigma) followed by immunoblotting with antibody specific for acetylated lysine (Cell Signaling Technology) or for Flag.

### Real-time PCR

Total RNA was isolated by using the TRIzol reagent extraction kit (Invitrogen), according to the manufacturer`s instructions. RNA was subsequently reverse transcribed to cDNA by using the high capacity cDNA archive kit (ABI). The mRNA levels were measured by real time PCR using the ABI PRISMTM 7900HT Sequence Detection System (Applied Biosystem).

### PDE assay

Phosphodiesterase activity was measured by modification of a previously published method (Manganiello and Vaughan, 1973) using 10 nM [^3^H]cAMP (45,000 cpm) or [^3^H]cGMP as substrates. Recombinant PDEs (1–5) from Signalchem were used for PDE activity assays in the presence of CBU90-93 (0–100 uM) or specific reference PDE inhibitors. Recombinant PDE1 (10 ng) activity was assayed by using 4 mg/ml calmodulin and 0.8 mM Ca^2+^ together with [^3^H]cAMP as substrate. Recombinant PDE2 (15 ng) activity was assayed in the presence of 1 mM cGMP, which increased PDE2 activity almost 3 fold. Activities of recombinant PDE3 (1 ng) and PDE4 (1 ng) were assayed by incubation in a reaction mixture containing 1 mg/ml BSA, with [^3^H]cAMP. Recombinant PDE5 (150 ng) activity was assayed using [^3^H]cGMP as substrate. PDE activity is expressed as pmol of cAMP or cGMP hydrolyzed/min/mg protein. The PDE3 reference inhibitor cilostamide and the PDE4 reference inhibitor rolipram were used to define the selectivity and potency of PDE inhibitors.

### Oxygen consumption measurements

Basal and palmitoleate stimulated oxygen consumption rate in C2C12 myotubes was measured following CBU91 incubation using a seahorse XFe24 analyser as previously described [14].

## Results and discussion

We screened new selective small molecules as potential inhibitors of PDE4. Our modular approach was based on two aromatic domains with a connector region (either amide or hydrazone). Design of initial structures was based on the ability of the molecule to access the PDE active site, diversity of structure, and modularity.

**Fig 1:**
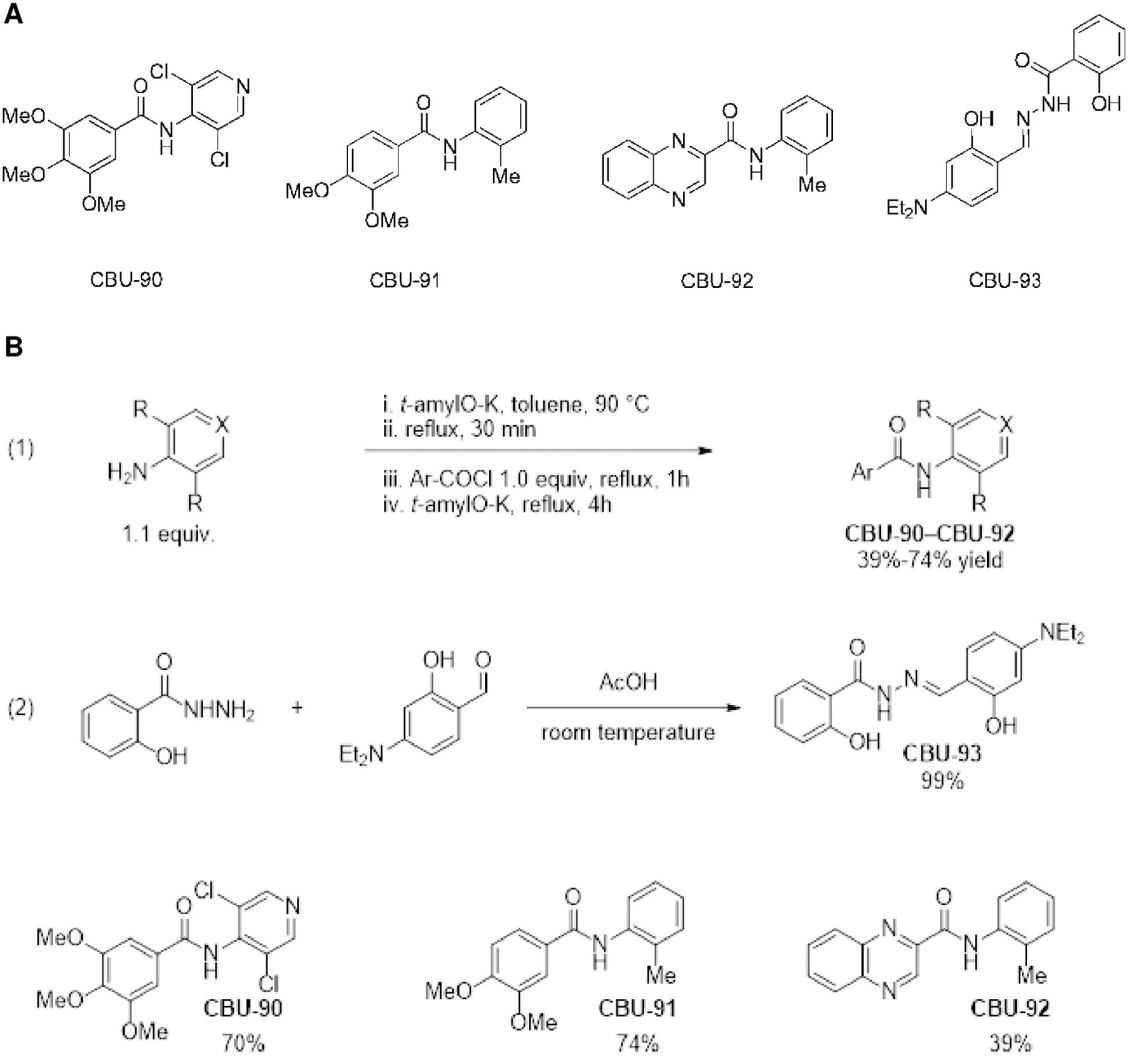
Initial group of small molecule inhibitors screened for activity against phosphodiesterases 1–5. A, Structure of CBU compounds (CBU90-93). B, Synthetic route of CBU90-93.

We tested four compounds, CBU90-93 (Fig 1), for their ability to inhibit five recombinant PDEs (PDE1-5).

**Fig 2:**
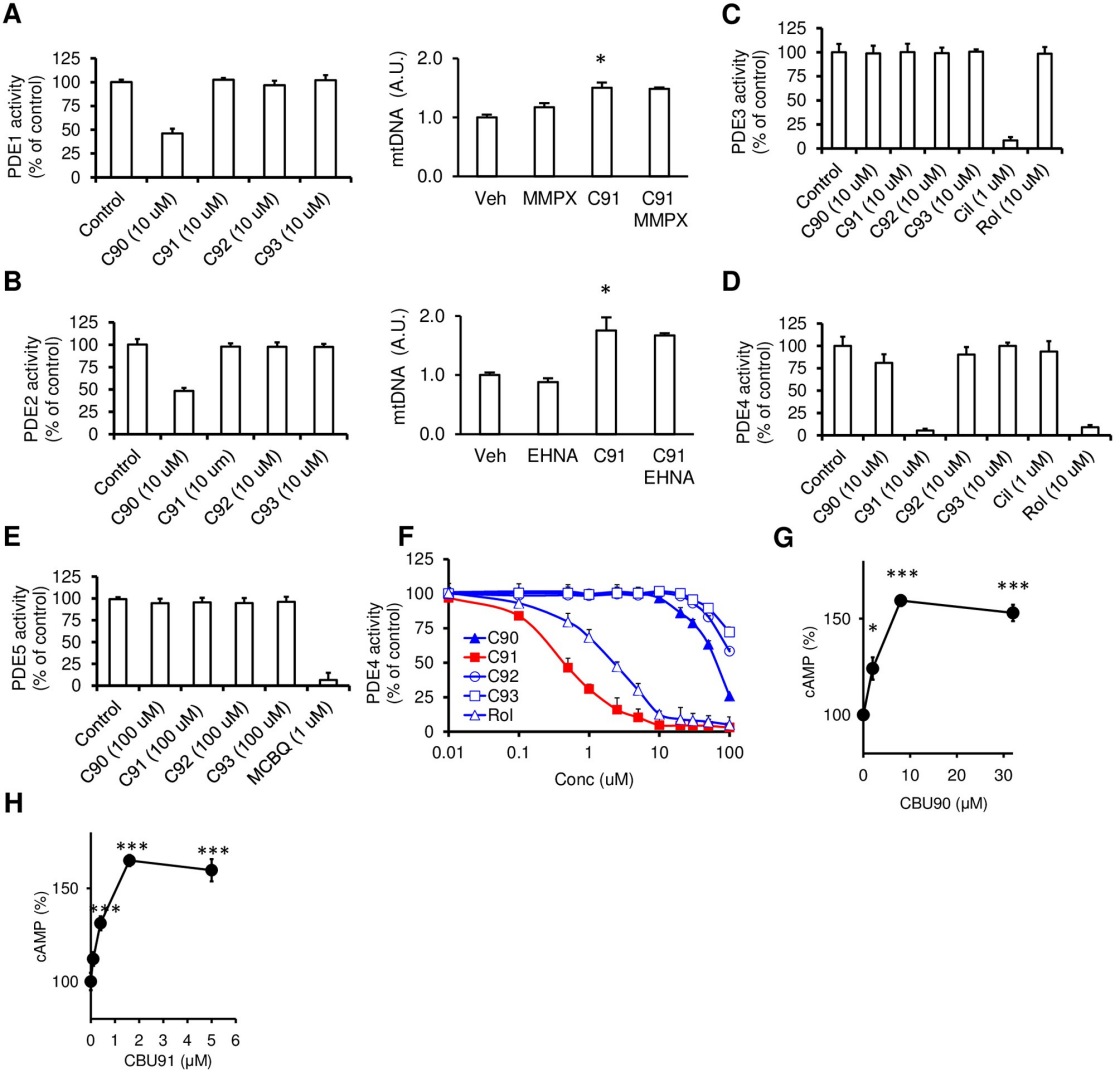
CBU91 is a specific PDE4 inhibitor. A-E, CBU compounds (C90-93) were screened at 10 μM against recombinant PDEs (1–5) using reference PDE inhibitors (PDE3 inhibitor, Cilostamide (Cil); PDE4 inhibitor, rolipram (Rol); PDE5 inhibitor MCBQ to define inhibitor selectivity and specificity. PDE1-4 activity was assayed as cAMP hydrolytic activity and PDE5 as cGMP hydrolytic activity. mtDNA content analyzed by means of quantitative PCR in C2C12 cells treated with CBU91 (10 μM) in the presence of PDE1 inhibitors (MMPX) or PDE2 inhibitor (EHNA) for 2 days (n = 3). F. Log concentration inhibition curves of CBU90-93 and a reference PDE4 inhibitor (rolipram). In vitro inhibition of PDE4D by CBU91 and rolipram demonstrated significantly lower IC50 values for CBU91 than rolipram. PDE activity was measured as pmol cAMP or cGMP hydrolysed/min/mg protein. A-F. Results are presented as % inhibition of PDE activities with recombinant PDE activities (1–5) taken as 100%. (n = 3). G, cAMP levels were measured in C2C12 myotubes with 0–30 μM CBU90 for 30 min (n = 3). H, cAMP levels were measured in C2C12 myotubes with 0–2 μM CBU91 for 30 min (n = 3). All values are expressed as mean ± s.e.m. *, p<0.05; **, p<0.005; ***, p<0.0005.

As shown in Fig 2, CBU92 and CBU93 had very weak PDE inhibitory activity, while CBU90 weakly inhibited PDEs 1, 2 and 4 with poor specificity, whereas CBU91 selectively inhibited PDE4. The PDE4 IC_50_ for CBU91 was significantly lower than rolipram (~0.45 μM vs. ~2.5 μM), indicating that CBU91 is a more potent PDE4 inhibitor than rolipram. In Fig 2A and 2B, we showed that pre-treatment with PDE1 inhibitors (MMPX) or the PDE2 inhibitor (EHNA) does not affect CBU91 induced mitochondrial biogenesis, indicating that CBU91 stimulated mitochondrial biogenesis is neither PDE1- nor PDE2-dependent.

In order to determine whether CBU91 exhibits PDE4 inhibitory activity *in vivo*, we treated C2C12 myotubes with varying concentrations of the potential inhibitor for 30 min and measured the intracellular cAMP concentration. We found that treatment with CBU90, the weaker PDE4 inhibitor, increased cAMP levels in a concentration-dependent manner, reaching saturation at ~8 μM (Fig 2G), whereas treatment with CBU91 increased cAMP levels in a concentration-dependent manner, but reached saturation at ~0.4 μM (Fig 2H).

**Fig 3:**
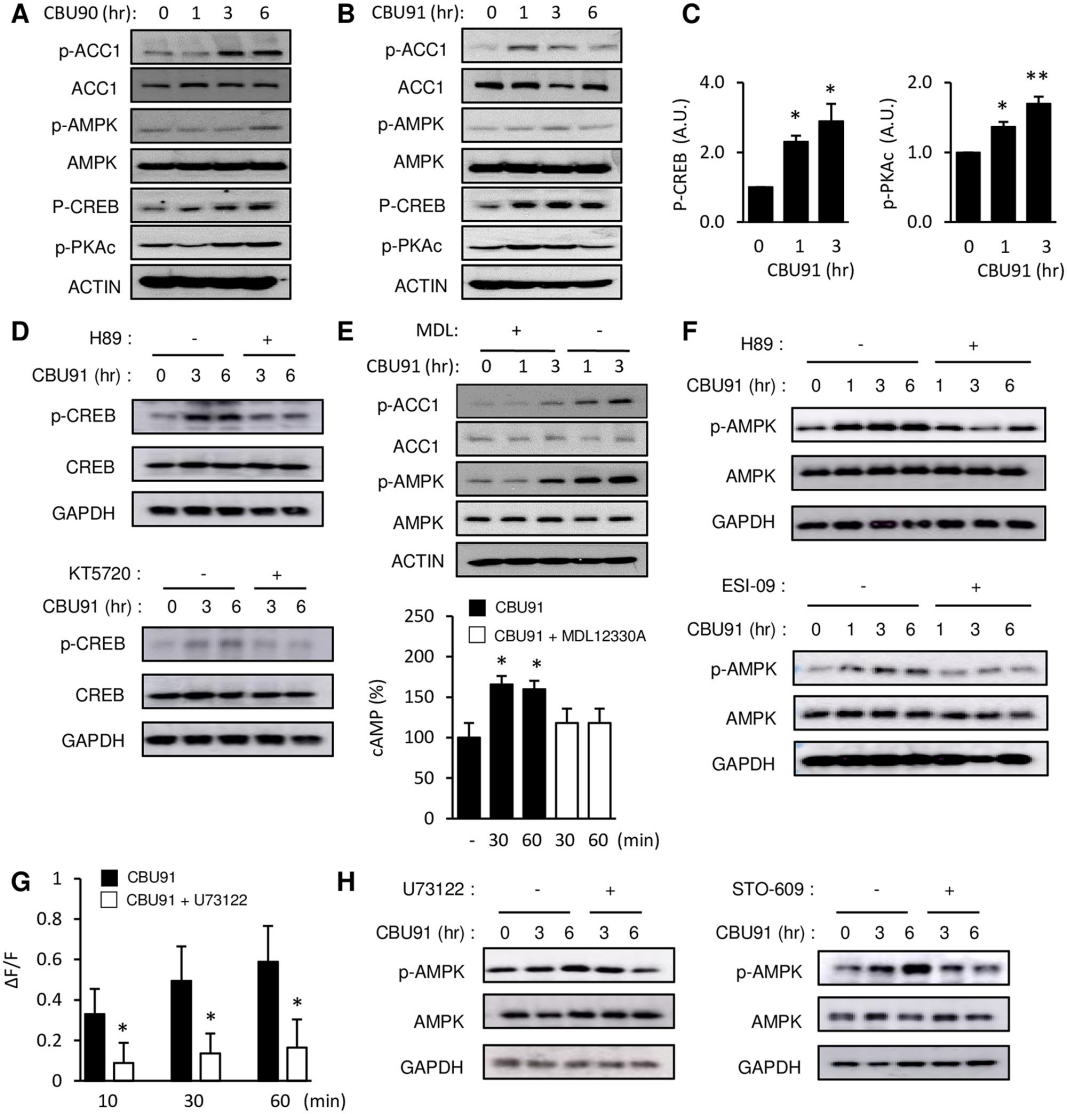
Compounds CBU90 and CBU91 activate AMPK via EPAC1. A, B, p-AMPK, p-ACC, p-CREB and p-PKAc in C2C12 myotubes treated with CBU90 and CBU91 in vitro are shown. C2C12 myotubes were treated with 20 μM CBU90 or 10 μM CBU91 for 1–6 hr. C, Quantification of PKA and CREB phosphorylation in C2C12 myotubes treated with CBU91 are shown (n = 3). D, p-CREB (S133) after treatment with CBU91(10 μM) in the presence of the PKA inhibitors (H89, KT5720). E, (Top) p-AMPK and p-ACC after treatment with CBU91 (10 μM) in the presence of the AC inhibitor MDL-12,330A in C2C12 myotubes. (Bottom) cAMP levels were measured in C2C12 myotubes with 10 μM CBU91 in the presence of the AC inhibitor MDL-12,330A (n = 5). F, p-AMPK after treatment with CBU91 (10 μM) in the presence of the PKA inhibitor (H89, 10 μM) or EPAC inhibitor (ESI-09, 10 μM). G, The increase in intracellular Ca2+ levels after CBU91 treatment (10 μM) in C2C12 myotubes loaded with Ca2+ indicator Fluo-4 AM in the presence of PLC inhibitor U73122 (20 μM). F indicates the fluorescence level and ΔF indicates the change in fluorescence (n = 5). H, p-AMPK after treatment with CBU91 (10 μM) in the presence of PLC inhibitor (U73122, 20 μM) or CamKK inhibitor (STO-609, 5 μg/ml). All values are expressed as mean ± s.e.m. *, p<0.05; **, p<0.005; ***, p<0.0005.

To confirm that the cAMP pathway is activated by these compounds in C2C12 myotubes, we performed immunoblotting with Thr197 phospho-specific antibody for protein kinase A (PKA), which is activated by cAMP [18] (Fig 3A and 3B). Phosphorylation of Ser133 in CREB, which is phosphorylated by PKA, was also visualized by immunoblotting and both phosphorylation events increased with CBU90 or CBU91 treatment (Fig 3A and 3B). Densitometric analysis showed that phosphorylation of PKA and CREB was significantly increased after CBU91 treatment (Fig 3C). Phosphorylation of CREB at S133 is observed with PKA but also with several other kinases. The pre-treatment with PKA inhibitors decreases CBU91 mediated CREB phosphorylation, indicating that CBU91 stimulated phosphorylation of CREB is PKA dependent (Fig 3D). The metabolic effects of PDE4 inhibition, such as increased mitochondrial content, protection against diet-induced obesity and improved glucose tolerance are AMPK-dependent [14]. To investigate the effect of CBU90 and CBU91 on AMPK activity, we performed immunoblotting with p-ACC (Ser79), which is phosphorylated by AMPK and with p-AMPK (T172) antibody, which is also commonly used as a readout of AMPK activity. As shown in Fig 3A and 3B, both CBU90 (20 μM) and CBU91 (10 μM) increased AMPK and ACC phosphorylation. Inhibiting cAMP production with the adenylyl cyclase inhibitor MDL-12,330A inhibited CBU91-mediated activation of AMPK (Fig 3E, Top) and blocked the CBU91-mediated increase in cAMP production (Fig 3E, Bottom), indicating that CBU91 activated AMPK in a cAMP-dependent manner. Consistent with this, inhibition of the cAMP signal transduction pathway by the PKA inhibitor or by the EPAC1 ((Exchange Protein Directly Activated by cAMP 1) inhibitor completely suppressed CBU91 mediated AMPK activation (Fig 3F). Since cAMP increases Ca^2+^ levels by way of phospholipase C (PLC) and EPAC1, we measured Ca^2+^ levels in the presence of the PLC inhibitor U73122. The CBU91-induced increase in intracellular Ca^2+^ was significantly reduced in the presence of the PLC inhibitor U73122 (Fig 3G). Consistent with this, U73122 prevented CBU91 from stimulating the phosphorylation of AMPK (Fig 3H, Left). Also, the Camkk inhibitor (STO-609) decreased the phosphorylation of AMPK after treatment with 10 μM CBU91, indicating that CBU91 stimulated the phosphorylation of AMPK in a CamKKβ-dependent manner (Fig 3H, Right).

**Fig 4:**
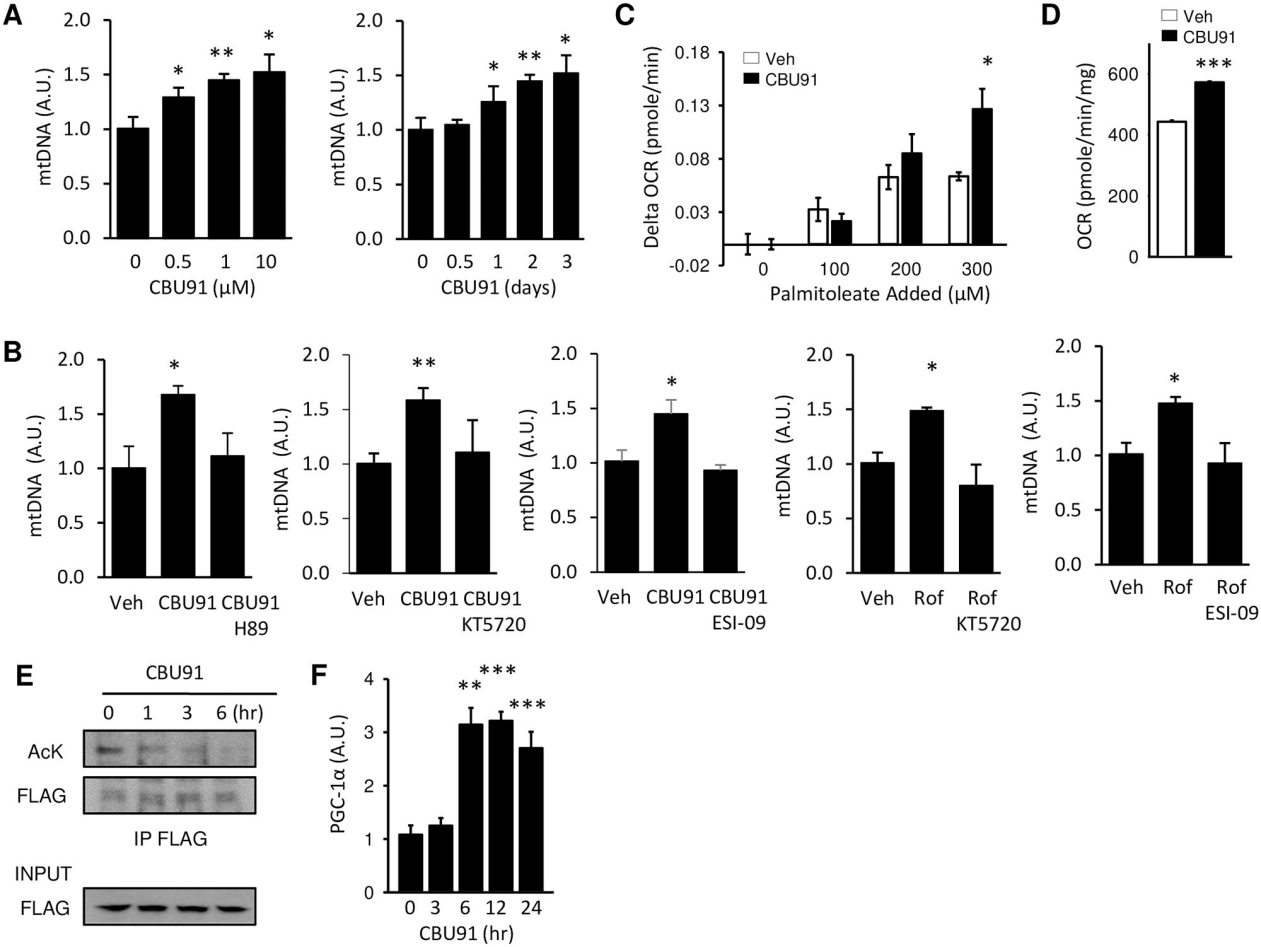
CBU91 increases mitochondrial biogenesis. A, Mitochondrial DNA content analyzed by means of quantitative PCR in C2C12 cells treated with 0–10 μM CBU91 for 2 days or 10 μM CBU91 for 0.5–3 days (n = 3). Relative expression values were normalized to untreated cells. B, Mitochondrial DNA content analyzed by means of quantitative PCR in C2C12 cells treated with CBU91 (10 μM) or roflumilast (10 μM) in the presence of PKA inhibitors (H89, KT5720) or EPAC inhibitor (ESI-09) for 2 days (n = 5). C, Change in oxygen consumption rate (Delta OCR) in C2C12 with palmitoleate injection (100–300 μM) after 6 hr treatment with CBU91. D, Oxygen consumption rate (OCR) in C2C12 myotubes 6 hr after treatment with CBU91. CBU91 pretreatment increases basal oxygen consumption rate (OCR) compared with control. E, To determine PGC-1α acetylation, we immunoprecipitated with an Flag antibody and immunoblotted for acetylated lysine (AcK) antibody in C2C12 myotubes treated with 10 μM CBU91 for 1–6 hr. F, The mRNA levels (arbitrary units) of PGC-1α in C2C12 myotubes after 5 μM CBU91 treatment for 3–24 hrs were measured by real-time PCR (n = 6).

Activation of AMPK is associated with an increase in mitochondrial biogenesis and function [19]. To study the effect of CBU91 on mitochondrial biogenesis, we measured mitochondrial DNA (mtDNA) copy number in C2C12 myotubes after treatment with CBU91. We found that CBU91 increased mtDNA copy number by ~50% after 2 days of treatment (Fig 4A). Also, pre-treatment with PKA inhibitors or the EPAC inhibitor suppressed both CBU91 and roflumilast induced mitochondrial biogenesis, indicating that CBU91 stimulated mitochondrial biogenesis in a cAMP-dependent manner (Fig 4B). One of the important functions of mitochondria is fat oxidation, an oxygen consuming process. Measurement of mitochondrial oxygen consumption in the presence of increasing concentrations of the fatty acid palmitoleate showed that CBU91 almost doubled the palmitoleate-induced oxygen consumption rate (OCR) (Fig 4C) and increased the total OCR by 29.2% (Fig 4D).

Rolipram has been shown to increase the activity of the protein deacetylase Sirt1, possibly by increasing the concentration of its cofactor NAD^+^ [14]. Sirt1 contributes to mitochondrial biogenesis by deacetylating the peroxisome proliferator-activated receptor-gamma coactivator-1α (PGC-1α), which is a mitochondrial regulator [20]. PGC-1α is a coactivator for its own transcription and as a result, activation of PGC-1α increases the mRNA levels of PGC-1α itself [21]. We examined whether CBU91 could increase PGC-1α deacetylation in C2C12 myotubes by immunoblotting with an antibody specific for acetylated lysine residues after immunoprecipitating PGC-1α. As shown in Fig 4E, CBU91 treatment decreased PGC-1α acetylation and consistent with its deacetylation/activation, expression of PGC-1α mRNA was also dramatically increased (Fig 4F). It has been reported that AMPK activates PGC-1α by direct phosphorylation [22] but PGC-1α phosphorylation by AMPK is a priming signal for subsequent deacetylation by SIRT1 [19]. Which suggests that CBU91 mediated AMPK activation increased mitochondrial biogenesis through the modulation of PGC-1α activity by SIRT1.

**Fig 5:**
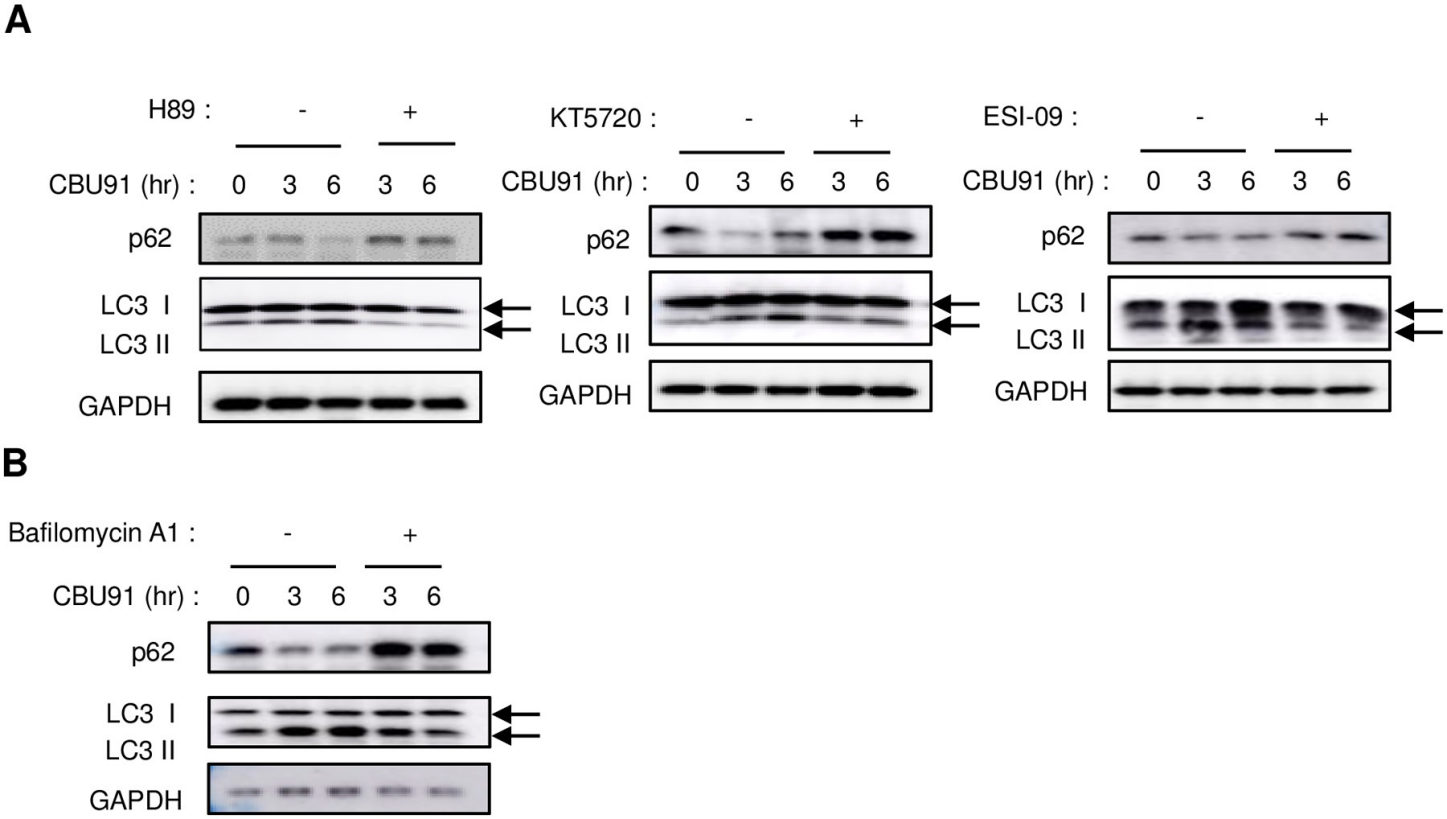
CBU91 increases autophagy. A, Expression level of LC3-I/II and p62 after treatment with CBU91 (10 μM) in the presence of the PKA inhibitors (H89, KT5720) or EPAC inhibitor (ESI-09). B, Expression level of LC3-I/II and p62 after treatment with CBU91 (10 μM) in the presence of the lysosomal inhibitor (Bafilomycin A1).

Autophagy is a highly conserved catabolic process. Organelles targeted for elimination are sequestered into lysosomes for degradation, and the resulting degradation products–amino acids, lipids, nucleic acids, carbohydrates–are released into the cell to support metabolic demands [23]. It has been reported that activation of AMPK induces autophagy [24]. To study the effect of CBU91 on autophagy, we examined the autophagy markers LC3 and p62. In CBU91 treated cells, the level of LC3II protein was gradually elevated, and the level of p62 was reduced, however, pre-treatment with PKA inhibitors, EPAC inhibitor and lysosomal inhibitor suppressed CBU91 induced autophagy (Fig 5A and 5B).

Since activation of AMPK can occur in the presence of apoptosis [25], we wanted to rule out the possibility that CBU91 was activating AMPK by inducing cell death. The potential effects of CBU91 on cell viability were tested in multiple ways. We used a CCK-8 proliferation assay, Hoechst 33342 staining and western blots (PARP, Caspase-3 cleavage) to determine the cell viability after CBU91 treatment. We observed no differences in cell viability between the CBU91 treated cells and the relevant control (data not shown).

Taken together, these findings indicate that CBU91 is a potent and selective PDE4 inhibitor that can activate AMPK and Sirt1 and improve mitochondrial function in myotubes. AMPK activation decreases glucose production, improves insulin sensitivity and decreases inflammation. Not surprisingly, AMPK is a highly sought-after target for treating metabolic diseases such as type-2 diabetes. Indeed, metformin, which activates AMPK indirectly in the liver, is the first-line drug for type-2 diabetes [26]. Recently, potent allosteric activators of AMPK have been developed to treat type-2 diabetes. Unfortunately, whole-body activation of AMPK led to cardiac enlargement, limiting its usefulness in humans [27]. Mutations of AMPK subunits in humans have been linked to cardiac enlargement [28], indicating that the cardiac effect of AMPK may be a class-wide effect of direct AMPK activators. On the other hand, PDE4 is not expressed in the human heart, and therefore, CBU91 should not have the same cardiac effect as the direct AMPK activators. Therefore, CBU91 may be an alternative to direct AMPK activators for treating metabolic diseases.

## Supporting information

S1 Raw image (TIF)

S2 Raw image (TIF)

S3 Raw image (TIF)

S4 Raw image (TIF)
