# Supplementary figures and images for: Potent PDE4 inhibitor activates AMPK and Sirt1 to induce mitochondrial biogenesis
Source: PLoS One. 2021 Jun 17;16(6):e0253269. doi: 10.1371/journal.pone.0253269 (PMC8211267; doi:10.1371/journal.pone.0253269)

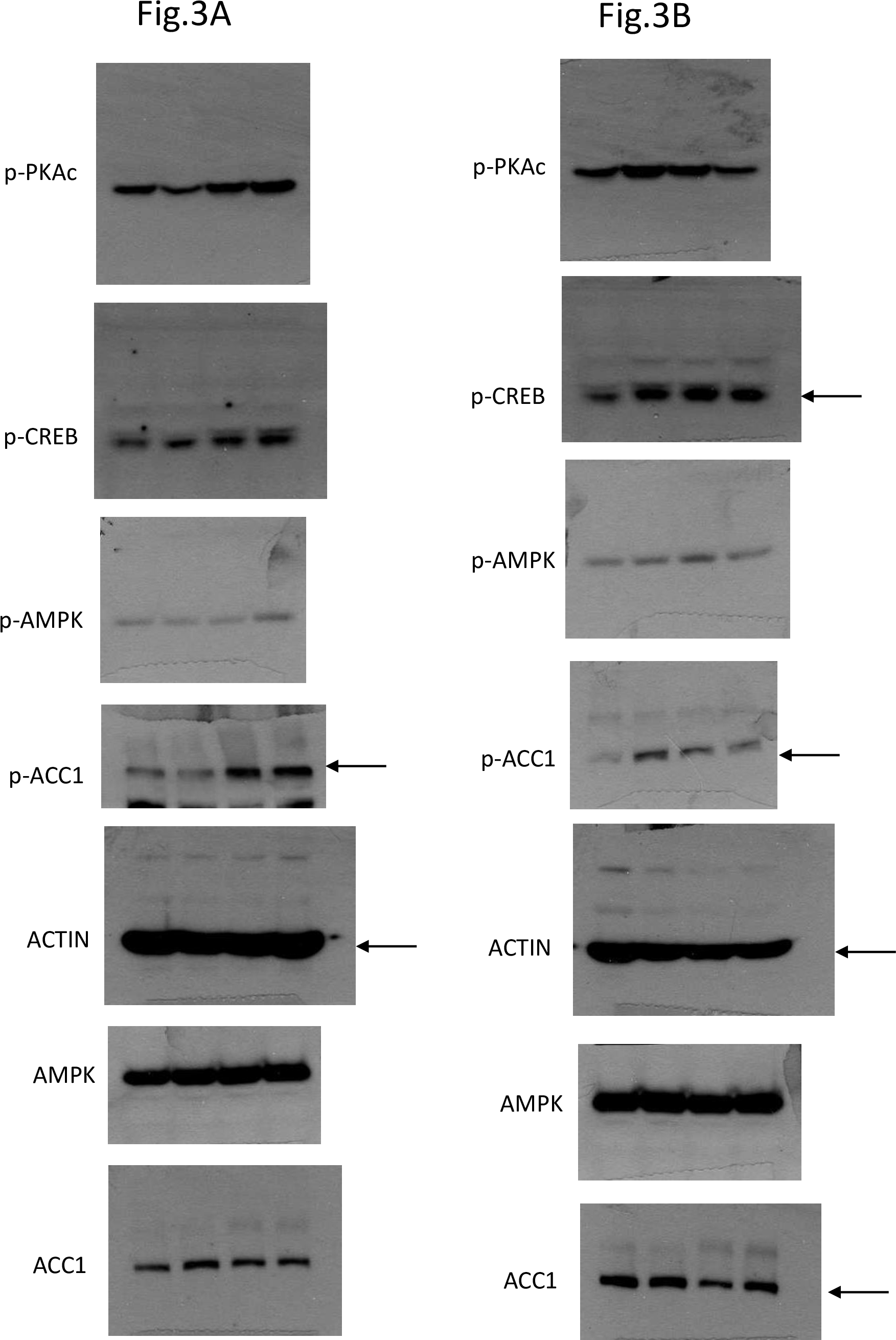

Supplement: S1 Raw image — (TIF) [file pone.0253269.s001.tif]

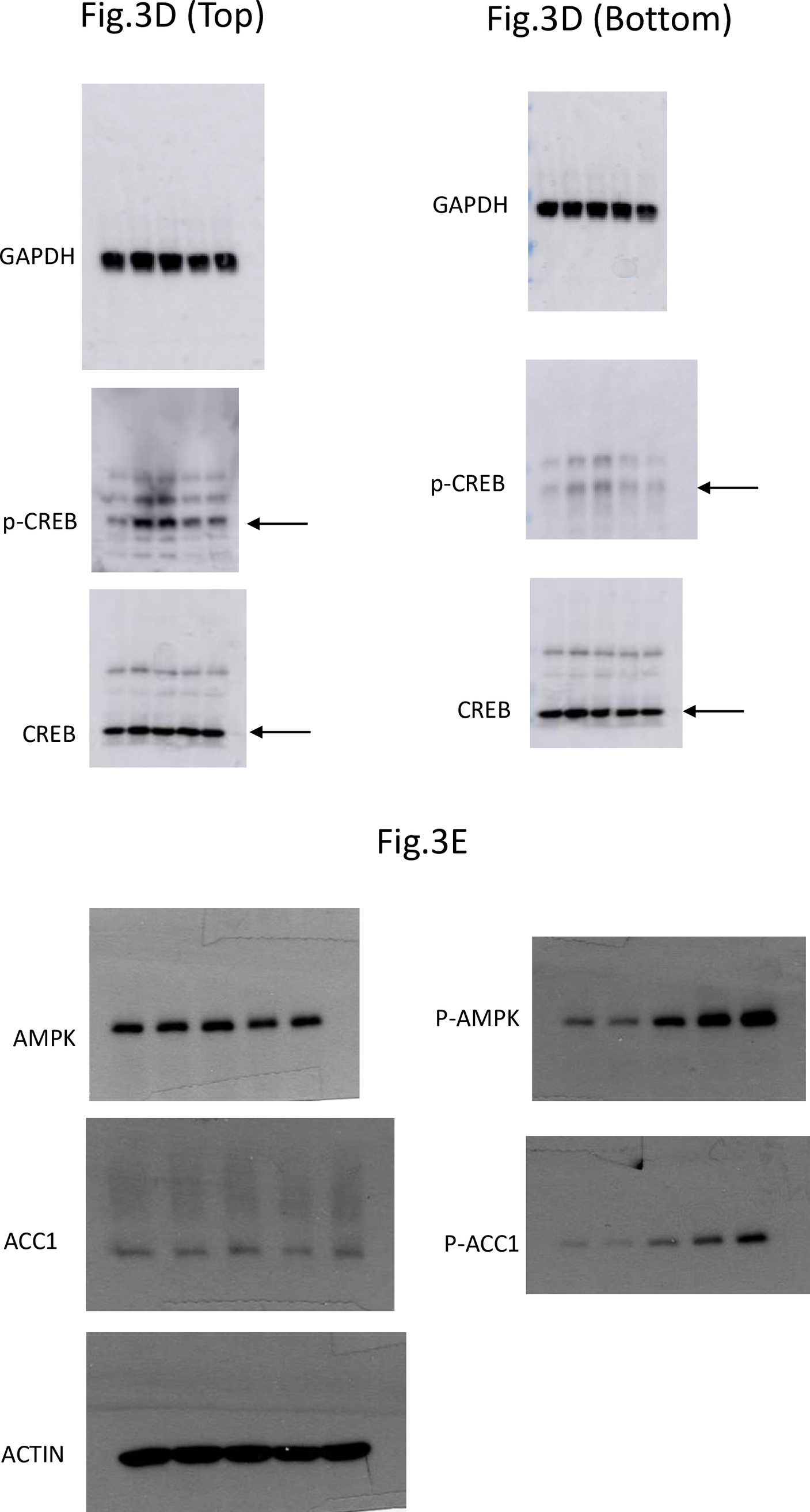

Supplement: S2 Raw image — (TIF) [file pone.0253269.s002.tif]

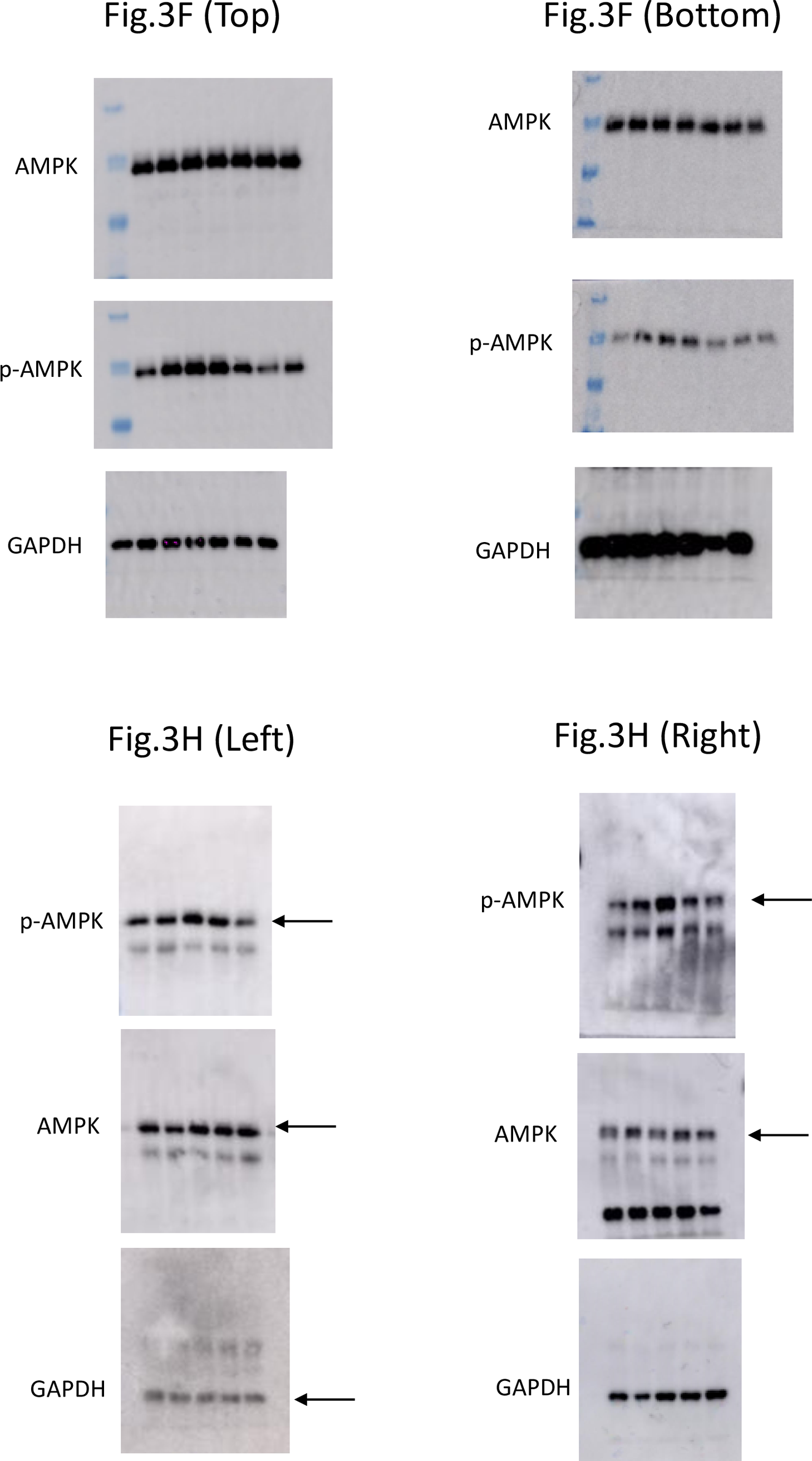

Supplement: S3 Raw image — (TIF) [file pone.0253269.s003.tif]

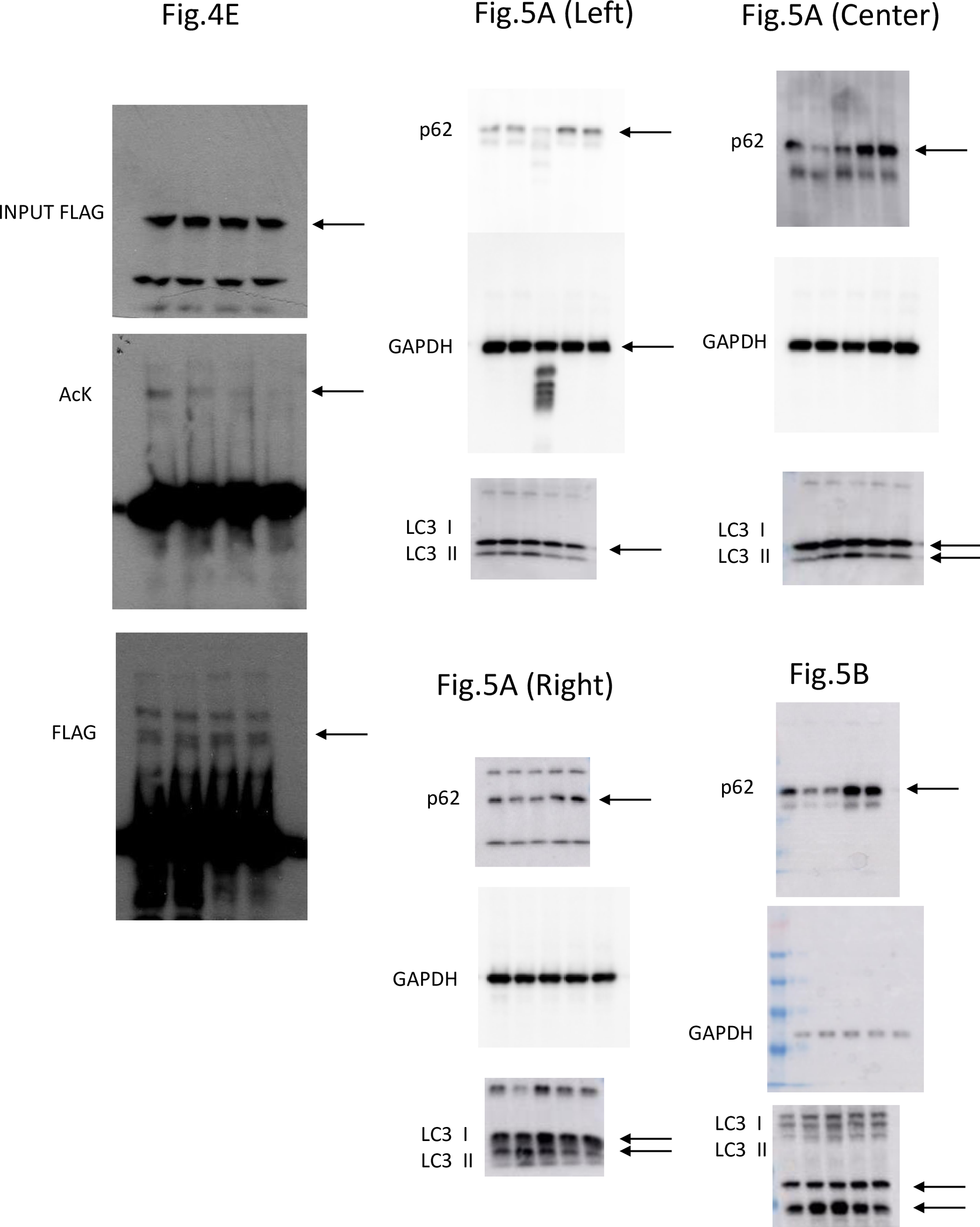

Supplement: S4 Raw image — (TIF) [file pone.0253269.s004.tif]
